# Steroid-Responsive Encephalopathy Associated With Autoimmune Thyroiditis Presenting With Parkinsonism

**DOI:** 10.7759/cureus.56184

**Published:** 2024-03-14

**Authors:** Guemouz Hicham, Yahya Naji, Wafa Hrouch, Sara Laadami, Nawal Adali

**Affiliations:** 1 Neurology Department, Agadir University Hospital, Agadir, MAR; 2 Neurology, Neurosciences Innovation Cognition Ethique (NICE) Research Team, Rein Endocrinologie Gastroentérologie Neurosciences Ethique (REGNE) Research Laboratory, Faculty of Medicine and Pharmacy, Ibn Zohr University, Agadir, MAR; 3 Neurology Department, University Hospital of Agadir, Agadir, MAR

**Keywords:** autoimmune thyroiditis, steroid‐responsive encephalopathy associated with autoimmune thyroiditis, ataxia, hashimoto's thyroiditis, parkinsonism

## Abstract

Steroid-responsive encephalopathy associated with autoimmune thyroiditis (SREAT) is a rare condition defined as encephalopathy with a positive antithyroid antibody. We report the case of a 52-year-old woman who presented with Parkinsonism associated with Hashimoto's thyroiditis. A few similar cases have been reported. Our patient responded well to corticosteroids with a significant reduction in symptoms. Diagnosis can pose a significant challenge in SREAT cases because of its variable clinical presentation. Therefore, we recommend evaluating thyroid function and thyroid autoantibodies in the context of acute and subacute encephalopathy. In the elderly population, SREAT, as a cause of Parkinsonism, should not be forgotten because of its simple treatment and significant improvements in neurological symptoms.

## Introduction

Steroid-responsive encephalopathy associated with autoimmune thyroiditis (SREAT) is a rare condition characterized by encephalopathy and the detection of antithyroid antibodies. The estimated prevalence of SREAT is 2.1 per 100,000, yet it remains underdiagnosed [1]. There is a female predominance, with an average age of onset of 40-55 years [2,3]. SREAT may manifest with highly variable manifestations, such as seizures, cognitive impairment, neuropsychiatric symptoms, stroke-like events, focal neurological deficit or movement disorders, and the presence of antithyroid peroxidase (TPO) antibodies and/or anti-thyroglobulin (TG) antibodies. It is more common in patients with normal or nonspecific results on both MRI and cerebrospinal fluid (CSF) analyses. Moreover, an increased level of antithyroid antibody titers and clinical corticosteroid responsiveness support the diagnosis [4]. To date, SREAT has rarely been associated with hypokinetic movement disorders. We describe a case of a progressive type of SREAT, primarily manifesting itself as parkinsonism.

## Case presentation

A 52-year-old right-handed woman with no known underlying disease, who was asymptomatic until one month prior to her admission to our neurology department, reported resting tremors affecting both upper limbs, more prominent on the left, syncopal episodes, and a depressive state.

Neurological examination revealed generalized rigidity with bradykinesia and exaggerated deep tendon reflexes in the four limbs. Moderate-to-severe truncal ataxia with bilateral dysmetria was observed. Eye movement examination revealed no square-wave jerks. A saccade examination revealed no slow or hypermetric saccades. There were no limitations to the range of eye movements in any direction. The cognitive assessment showed mild deficits in attention, with no abnormalities in visuoperceptual executive function or personality. The thyroid gland, lymph nodes, and overall examination were all normal.

Laboratory tests included normal complete blood cell counts, electrolytes, renal and hepatic function, uric acid, hepatitis B and C, HIV serologies, coagulation parameters, protein levels, protein electrophoresis, vitamin B1, B6, B9, B12, and immunological panels, namely, anti-DNA antibodies, anti-Sjögren's syndrome A (anti-SSA), anti-Sjögren's syndrome B (anti-SSB), and antineutrophil cytoplasmic antibodies (ANCA). Examination of the cerebrospinal fluid (CSF) revealed a protein level of 0.37 g/l without pleocytosis and no oligoclonal bands. Thyroid function tests revealed elevated thyroid-stimulating hormone (TSH) levels (12 micro-IU/ml). Serum levels of anti-thyroid peroxidase antibody (anti-TPOAb; 138 IU/ml, range: <60 IU/ml) and thyroglobulin antibody (anti-TGAb; 38 IU/ml, range: <10 IU/ml). CSF anti-TPOAb levels were not measured.

**Figure 1:**
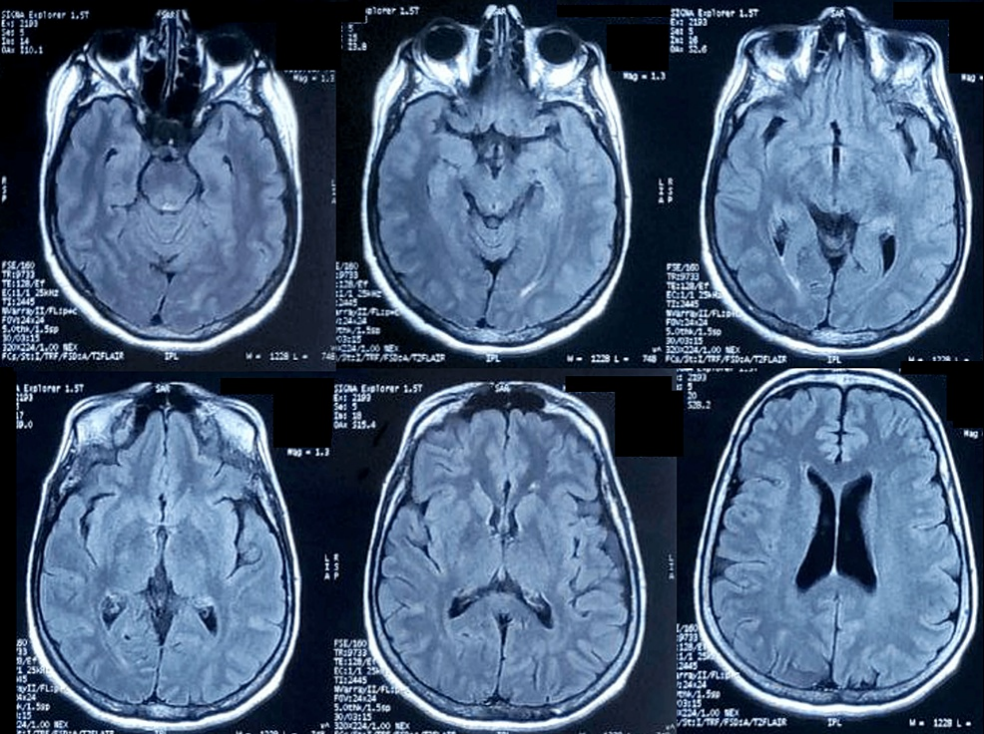
Brain MRI with axial and FLAIR sequences Absence of cortical or white matter signal abnormality, with no lesion in the basal ganglia and the midbrain. FLAIR - fluid-attenuated inversion recovery

**Figure 2:**
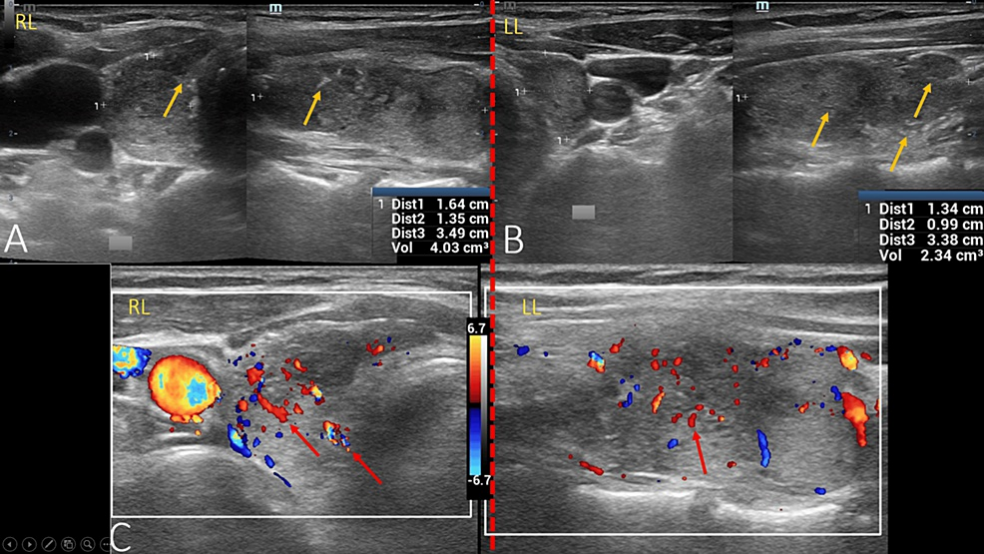
Thyroid ultrasound images A: atrophic right thyroid lobe (RL), heterogeneous hypoechoic echostructure with a few hyperechoic septa, producing a pseudo-nodular appearance (yellow arrows). B: atrophic left thyroid lobe (LL), with heterogeneous hypoechoic echostructure due to the presence of echogenic punctuations (yellow arrows). C: hypervascularization aspect on color Doppler of both thyroid lobes (red arrows).

No abnormalities were observed on cerebral MRI (Figure 1) and full-body CT scan. Moreover, signs of chronic atrophic thyroiditis were observed on ultrasonography (Figure 2).

Based on the clinical picture, subacute parkinsonism, elevated anti-thyroid antibodies, and after ruling out vascular, metabolic, infectious, and neoplastic conditions, a diagnosis of parkinsonism due to SREAT was suggested, and corticosteroid therapy was initiated. Intravenous treatment with a high dose of methylprednisolone 1000 mg over five days was provided and well tolerated, relayed by 60 mg orally with slow progressive tapering (10 mg every 15 days for approximately six months), along with dopamine therapy and fluoxetine. Follow-up showed normalization of thyroid function with a marked reduction in the severity of parkinsonism. The Unified Parkinson's Disease Rating Scale (UPDRS) motor score improved by 40% six months later.

## Discussion

Hashimoto's encephalopathy (HE) was first reported in 1966 by Brain et al.; he described a case of a 63-year-old man who had manifested signs of encephalopathy with confusion and epileptic seizures associated with recurrent hemiparesis; the CSF analysis revealed hyperproteinorachia and the thyroid biopsy confirmed Hashimoto's thyroiditis [4]. The prevalence is estimated to be 2.1/100,000, with an average starting age of 45 years (12-84 years). The sex ratio was four women per man [5]. There is no typical clinical presentation, and the clinical manifestations most frequently associated with cognitive and behavioral disorders are fine tremors, language impairment, headaches, and sleep disorders [1,6]. The onset of symptoms can be acute, progressive, or recurrent. Some authors describe two clinical forms of SREAT: vascular, associated with stroke, epileptic seizures, and psychomotor retardation, and psychiatric, with confusion, hallucinations, and psychotic state. Switching from one form to another is possible [2]. Children can also be affected; clinical features are variable, but the most reported manifestations are disorientation, hallucinations, and epileptic seizures associated with progressive cognitive decline [7]. Although various hypotheses favor an autoimmune mechanism, the pathophysiology of this disease remains unclear. The high levels of thyroid antibodies in the blood and CSF and the excellent response to corticosteroids are strong arguments in favor of the autoimmune nature of the disease [5]. The autoimmune mechanism is also supported by the discovery of autoantibodies directed against the N-amino-terminal end of alpha-enolase [4]. The direct toxic role of thyroid hormones in the brain has never been verified [8].

Our patient presented with subacute onset of parkinsonism with cerebellar ataxia and depression. Movement disorders can manifest as SREAT, and the common phenomena are myoclonus, tremors, and ataxia [9]. Parkinsonism is less commonly reported in these rare conditions [10]. Although there is a correlation between thyroid function and parkinsonism without encephalopathy, the underlying mechanism is not completely clear [10-11]. When parkinsonism is present as a predominant feature, the diagnosis can easily be missed or mistaken for degenerative causes.

Complementary examinations are mandatory for diagnosis, especially in the presence of elevated serum levels of antithyroid antibodies [12]. Occasionally, titers of antithyroid antibodies are obtained from the CSF, while no strong association between the severity of neurological symptoms and serum or CSF concentration of antibody titers has been established [13]. Our patient had elevated serum levels of anti-TPOAb and anti-TGAb. Earlier studies show that 17-20% of patients are hypothyroid, 7% are hyperthyroid, and 38-47% of patients are euthyroid [12], our patient was hypothyroid. EEG can show non-specific slow background activity [12]. Brain imaging, including CT and MRI scans, showed no abnormalities, and the same results are seen in more than 50% of SREAT cases [13,14].

Although this disorder has long been reported in the literature, several controversial issues persist, including the wide spectrum of its clinical features, unclear association with thyroid disorders, and pathogenic roles of anti-TPOAb and anti-TGAb, as well as their correlations with clinical severity [10,11,15]. In addition, studies have suggested that the presence of thyroid autoantibodies (antithyroid peroxidase and antithyroglobulin) is associated with neurodegenerative disorders, such as multiple system atrophy (MSA) and cerebellar degeneration [11].

The most common treatment for HE is the administration of a high dose of corticosteroids, 1 g daily of methylprednisolone for three to seven days, relayed by oral prednisone 1-2 mg/kg/day for six to eight weeks, followed by a gradual decrease in doses [16]. Generally, improvement is obtained within 72 hours, especially in confusional states. Other immunosuppressive therapies, including methotrexate, azathioprine, cyclophosphamide, and mycophenolate mofetil, have been used sporadically during corticosteroid complications and resistant cases [17].

## Conclusions

Few studies have shown a correlation between thyroid dysfunction and parkinsonism, but the underlying mechanism is unclear. Further investigations should be conducted to provide clear evidence of this association. As SREAT is a treatable condition, rapid and accurate diagnosis is needed in all patients with hypo- or hyperkinetic movement disorders.
